# Gut microbiota dysbiosis: Pathogenic drivers and emerging phage-based therapeutics

**DOI:** 10.3934/microbiol.2026028

**Published:** 2026-09-11

**Authors:** Ahmed Dewan, Débora Brito Goulart, Maria Teresa Mascellino

**Affiliations:** 1 College of Life Sciences, Anhui Agriculture University, Hefei 230036, China; 2 Department of Veterinary Microbiology and Preventive Medicine, Iowa State University (2182 Vet Med, Ames, IA 50011 -USA; 3 Department of Public Health and Infectious Diseases, Sapienza University of Rome, 00185 Rome, Italy

**Keywords:** gut microbiota, dysbiosis, pathogenic bacteria, bacteriophage (phage) therapy, fecal microbiota transplantation, live biotherapeutic products, precision microbiome medicine

## Abstract

Gut microbiota dysbiosis driven by pathogenic and opportunistic bacteria, including *Clostridioides difficile*, adherent-invasive *Escherichia coli* (AIEC), *Salmonella enterica serovar Typhimurium*, *Fusobacterium nucleatum*, *Klebsiella pneumoniae*, *Enterococcus faecalis*, and *Campylobacter* spp., disrupts colonization resistance, epithelial barrier integrity, and immune homeostasis, thereby contributing to the development of inflammatory and metabolic diseases. In this review, we summarize the mechanisms through which these microorganisms promote gut dysbiosis and critically evaluate current microbiome-targeted therapeutic strategies, including fecal microbiota transplantation (FMT), live biotherapeutic products (LBPs), probiotics, and bacteriophage (phage) therapy. We review the preclinical and emerging clinical evidence supporting phage-based targeting of major dysbiosis-associated pathogens and examine the principal challenges limiting clinical translation, including phage resistance, immune-mediated clearance, safety considerations related to temperate phages, manufacturing and delivery constraints, and regulatory barriers. Although evidence remains predominantly preclinical, advances in phage engineering, artificial intelligence-guided phage selection, and systems-level microbiome modeling are expanding the potential of personalized phage therapeutics. Collectively, these developments position bacteriophage therapy as a promising addition to future microbiome-directed strategies for restoring gut microbial homeostasis and managing dysbiosis-associated disease.

## 1. Introduction

The human gut microbiota is a highly complex and dynamic ecosystem comprising bacteria, archaea, fungi, protozoa, and viruses that collectively contribute to host health through nutrient metabolism, immune regulation, and maintenance of epithelial barrier integrity [1]. Based on estimates for a 70-kg reference adult male, which contains approximately 3.8 × 10^13^ (38 trillion) bacterial cells [2]. Disruption of this ecological balance, termed dysbiosis, is characterized by reduced microbial diversity, altered community composition, and impaired microbial functionality, ultimately compromising gut homeostasis [3]–[5]. Dysbiosis has been associated with chronic disorders, including obesity, insulin resistance, type 2 diabetes, cardiovascular disease, chronic liver disease, inflammatory bowel disease (IBD), colorectal cancer (CRC), and neuropsychiatric conditions such as anxiety and depression [6]–[8]. Furthermore, disruption of intestinal barrier integrity facilitates the translocation of microbial-derived products, including lipopolysaccharides (LPSs) and other pro-inflammatory microbial components, into the systemic circulation, promoting intestinal permeability (“leaky gut”), chronic inflammation, and disease progression [9]. These findings highlight the pivotal role of gut microbiota homeostasis in maintaining human health and underscore dysbiosis as a key contributor to the initiation and progression of many inflammatory and metabolic diseases.

The gut virome consists primarily of bacteriophages (phages) and eukaryotic viruses, representing a dynamic and integral component of the intestinal microbiome [10]. The virome contributes to intestinal homeostasis by regulating bacterial population dynamics, facilitating horizontal gene transfer, and maintaining microbial diversity metabolism and immune responses [10],[11]. Phages can selectively modulate the abundance and function of commensal and pathogenic bacteria, thereby influencing microbial community structure and metabolic activity [12]. In addition, interactions between the gut virome and the host immune system play a critical role in mucosal immunity, promoting immune maturation, and maintaining tolerance to commensal microorganisms while enhancing defense against invading pathogens [13]. Alterations in virome composition, often referred to as viral dysbiosis, have been associated with IBD, CRC, and other chronic inflammatory conditions, suggesting that a balanced virome is essential for preserving intestinal health and ecosystem stability [14],[15].

Within the range of factors contributing to gut dysbiosis, pathogenic bacteria are recognized as major ecological drivers capable of disrupting microbial homeostasis through multiple mechanisms, including epithelial barrier damage, immune activation, metabolic remodeling, toxin production, and competitive exclusion of beneficial microorganisms [16]–[20]. In this review, we focus on the most extensively studied pathogens implicated in gut dysbiosis and associated diseases such as *Clostridioides difficile*, adherent-invasive *Escherichia coli* (AIEC), *Salmonella Typhimurium*, *Fusobacterium nucleatum*, *Klebsiella pneumoniae*, *Enterococcus faecalis*, and *Campylobacter* spp. These microorganisms contribute to dysbiosis through diverse mechanisms, including epithelial barrier disruption, chronic inflammation, oxidative stress, production of toxic metabolites, alteration of microbial community structure, and promotion of tumorigenesis [16]–[20]. Among currently available microbiome-targeted interventions, we are focusing on fecal microbiota transplantation (FMT), live biotherapeutic products (LBPs), probiotics, and phage therapy represent distinct yet complementary strategies for restoring microbial homeostasis.

FMT involves the transfer of a complex microbial community from a healthy donor to a recipient and has demonstrated remarkable efficacy in the treatment of recurrent *C. difficile* infection (rCDI) [21],[22]. In contrast, LBPs consist of defined strains of live microorganisms developed to confer specific therapeutic benefits. LBPs offer greater reproducibility, improved safety profiles, and enhanced regulatory control owing to their standardized composition [23],[24]. Phage therapy differs fundamentally from both approaches by selectively targeting pathogenic bacteria while maintaining the beneficial commensal microorganisms, thereby minimizing collateral disruption of the resident microbiota [25],[26]. Moreover, while FMT and LBPs primarily seek to restore microbial equilibrium, phage therapy aims to eliminate specific disease-associated bacterial populations that drive dysbiosis [27],[28]. This high degree of specificity positions phages as promising precision microbiome therapeutics, highlighting their potential as either standalone interventions or complementary tools alongside established microbiome restoration strategies [26]–[28].

In this review, we aim to: (i) summarize the mechanisms through which key dysbiosis-associated bacteria, including *C. difficile*, adherent-invasive *Escherichia coli* (AIEC), *Salmonella Typhimurium*, *F. nucleatum*, and other clinically relevant pathobionts, disrupt gut microbial homeostasis; (ii) critically evaluate current microbiota-targeted therapeutic approaches, including FMT, LBPs, probiotics and bacteriophages; (iii) compare the advantages, limitations, and therapeutic potential of these interventions for restoring microbial balance; and (iv) discuss the challenges and future perspectives.

## 2. Gut dysbiosis

Gut dysbiosis is an imbalance in the composition, diversity, and functional activity of the intestinal microbiota that disrupts host–microbe homeostasis [4]. It is marked by the depletion of beneficial microorganisms, expansion of opportunistic pathogens, and altered microbial metabolic function, and it has been linked to impaired intestinal barrier integrity, immune dysregulation, and chronic inflammation, all of which drive gastrointestinal disorders [4],[29]. Antibiotic exposure, diet, infection, and environmental stress can each disrupt the ecological balance of the gut microbial community and induce dysbiosis [5].

### 2.1. Pathogenic bacteria-induced gut dysbiosis

Many bacterial species have been implicated in dysbiosis-related disorders, and several pathogens have emerged as representative models for understanding the diverse mechanisms underlying microbiota disruption. In the following sections, we discuss the major pathways through which these pathogens promote gut dysbiosis and contribute to disease pathogenesis.

#### 2.1.1. Clostridioides difficile

*C. difficile* is a well-studied example of pathogen-associated gut dysbiosis; it shows how disruption of the resident microbiota opens the door to pathogen colonization and disease [30]. As shown in Figure 1, *C. difficile* exploits the loss of colonization resistance that follows disturbance of the gut microbial ecosystem, particularly after exposure to broad-spectrum antibiotics [30]–[33]. A key mechanism is altered bile acid metabolism; depletion of commensal bacteria reduces conversion of primary bile acids into secondary bile acids, so increased primary bile acids (particularly taurocholate) promote spore germination, while reduced inhibitory secondary bile acids let vegetative growth and colonization proceed [30]–[33].

Once colonized, *C. difficile* produces its major virulence factors, toxins A (TcdA) and B (TcdB), which translocate into the cytosol of target cells and inactivate small GTP-binding proteins, including RhoA, Rac, and Cdc42 [34],[35]. This disrupts actin cytoskeleton organization, causing loss of tight-junction integrity, increased epithelial permeability, cell rounding, and epithelial cell death [34]–[36]. Barrier disruption enables microbial products to translocate and promotes the recruitment of inflammatory cells, resulting in a pronounced inflammatory response and mucosal injury [34]–[36].

In hypervirulent strains, the binary toxin CDT contributes to pathogenesis by inducing microtubule-based protrusions that promote bacterial adherence and colonization of the intestinal epithelium [37]. This can contribute to a self-perpetuating cycle in which toxin-mediated epithelial damage and inflammation further disrupt microbial homeostasis, impair the recovery of beneficial commensal populations, and promote persistent or recurrent dysbiosis.

**Figure 1.**
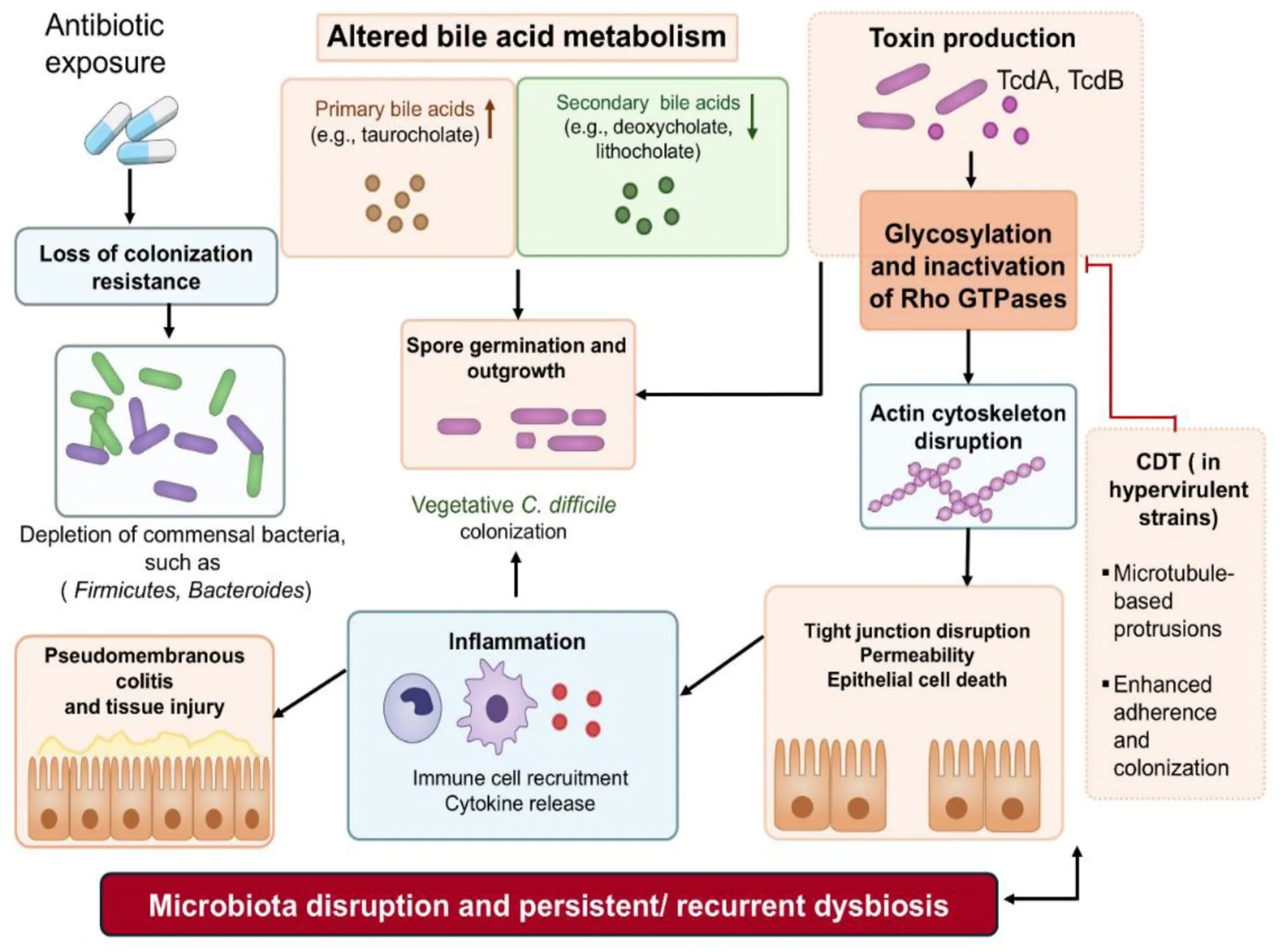
Mechanism of *Clostridioides difficile* in gut dysbiosis. *C. difficile* exploits disruptions in the gut microbiota, particularly following antibiotic exposure, which reduce colonization resistance and alter bile acid metabolism [32],[38],[39]. The resulting increase in primary bile acids and depletion of inhibitory secondary bile acids promote spore germination and bacterial expansion [33],[39]. Following colonization, *C. difficile* produces toxins A (TcdA) and B (TcdB), which inactivate Rho-family GTPases, leading to actin cytoskeletal disruption and cell rounding, loss of tight-junction integrity, increased epithelial permeability, and epithelial cell death. These events trigger inflammatory responses and tissue injury, culminating in pseudomembranous colitis and persistent dysbiosis [34]–[37].

#### 2.1.2. *Salmonella* enterica serovar *Typhimurium*

*Salmonella* enterica serovar *Typhimurium* (*S. Typhimurium*) is a facultative intracellular pathogen that contributes gut dysbiosis largely through inflammation-driven disruption of colonization resistance and intestinal nutrient ecology [40]–[43]. As shown in Figure 2, infection begins with bacterial adhesion to intestinal epithelial cells and activation of the SPI-1-encoded type III secretion system (T3SS), which translocates effectors such as SopE, SopE2, and SopB into host cells. These effectors manipulate Rho-family GTPases and cytoskeletal pathways, promoting membrane ruffling, bacterial uptake, and formation of *Salmonella*-containing vacuoles (SCVs) [40]–[43].

**Figure 2.**
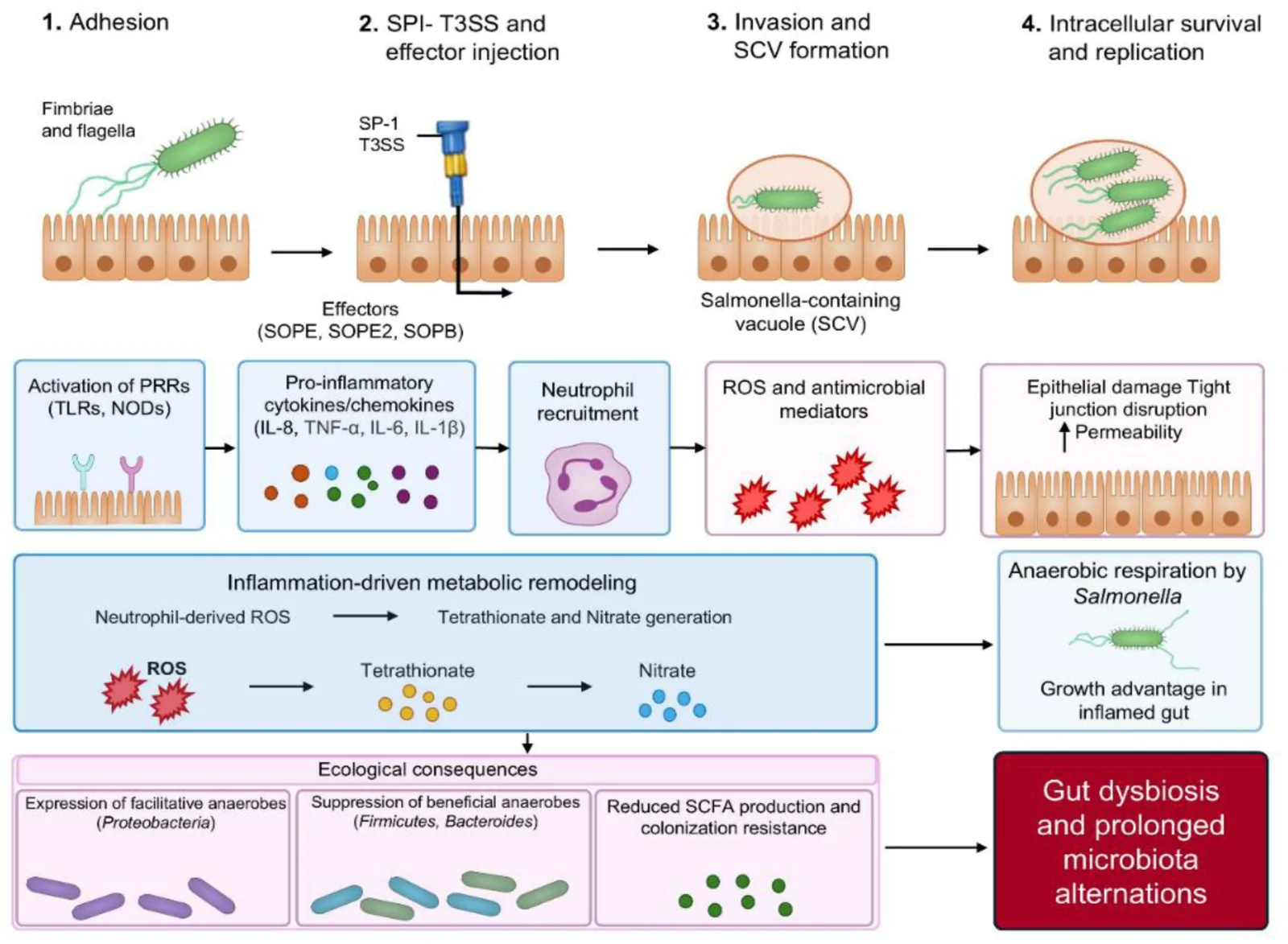
Mechanism of *Salmonella Typhimurium* in gut dysbiosis. *S. Typhimurium* initiates infection by adhering to intestinal epithelial cells and delivering virulence effectors through the SPI-1 type III secretion system (T3SS). These effectors manipulate host signaling and cytoskeletal pathways to promote bacterial uptake and formation of *Salmonella*-containing vacuoles (SCVs). Infection triggers mucosal inflammatory responses, including the production of pro-inflammatory cytokines and chemokines and recruitment of neutrophils, which can contribute to epithelial injury and barrier dysfunction through reactive oxygen species (ROS) and other antimicrobial mediators [42],[44],[45]. In parallel, inflammation alters the intestinal metabolic environment by generating alternative electron acceptors such as nitrate and tetrathionate, enabling *S. Typhimurium* to gain a growth advantage over many obligate anaerobic commensals and thereby promote gut dysbiosis [40],[41],[46],[47].

Invasion triggers a strong inflammatory response characterized by the production of pro-inflammatory cytokines and chemokines, such as IL-8, TNF-α, IL-6, and IL-1β, which recruits neutrophils and other immune cells to the site of infection [44],[45],[48],[49]. Activated neutrophils release ROS and antimicrobial mediators that injure the epithelium, disrupt barrier integrity, and increase intestinal permeability [44],[45],[48],[49]. Although these responses are intended to restrict bacterial growth, they can also reshape the intestinal environment and promote dysbiosis. A key mechanism underlying *S. Typhimurium*-induced dysbiosis is its ability to exploit inflammation-associated metabolic changes in the gut. Inflammation and neutrophil-derived ROS contribute to the generation of alternative electron acceptors, including tetrathionate and nitrate, which *S. Typhimurium* can use for anaerobic respiration [46],[47],[50], providing it a competitive advantage over many obligate anaerobic commensals. This inflammation-driven expansion of *S. Typhimurium* can suppress beneficial microbial populations and promotes the expansion of Enterobacteriales, contributing to persistent dysbiosis [51]–[53].

#### 2.1.3. Adherent-Invasive *Escherichia coli* (AIEC)

Adherent-invasive *Escherichia coli* (AIEC) is a pathobiont strongly associated with IBD, particularly Crohn's disease, and is recognized as an important contributor to inflammation-associated gut dysbiosis [54]–[56]. As illustrated in Figure 3, AIEC initiates colonization through adhesins that mediate attachment to intestinal epithelial cells via host receptors [54]–[56]. Following attachment, AIEC invades epithelial cells and survives intracellularly, enabling persistence within the intestinal mucosa and facilitating prolonged interactions with host tissues [54]–[56].

A central driver of AIEC-induced dysbiosis is the activation of inflammatory signaling. LPSs activate Toll-like receptor 4 (TLR4), while other bacterial components stimulate additional pattern-recognition receptors, leading to NF-κB activation and the production of pro-inflammatory cytokines [56]–[59]. These mediators recruit immune cells and amplify mucosal inflammation [60],[61]. AIEC can also survive and replicate within macrophages while sustaining cytokine production, particularly TNF-α, thereby contributing to chronic intestinal inflammation and facilitating pathogen persistence within the host [60],[61].

**Figure 3.**
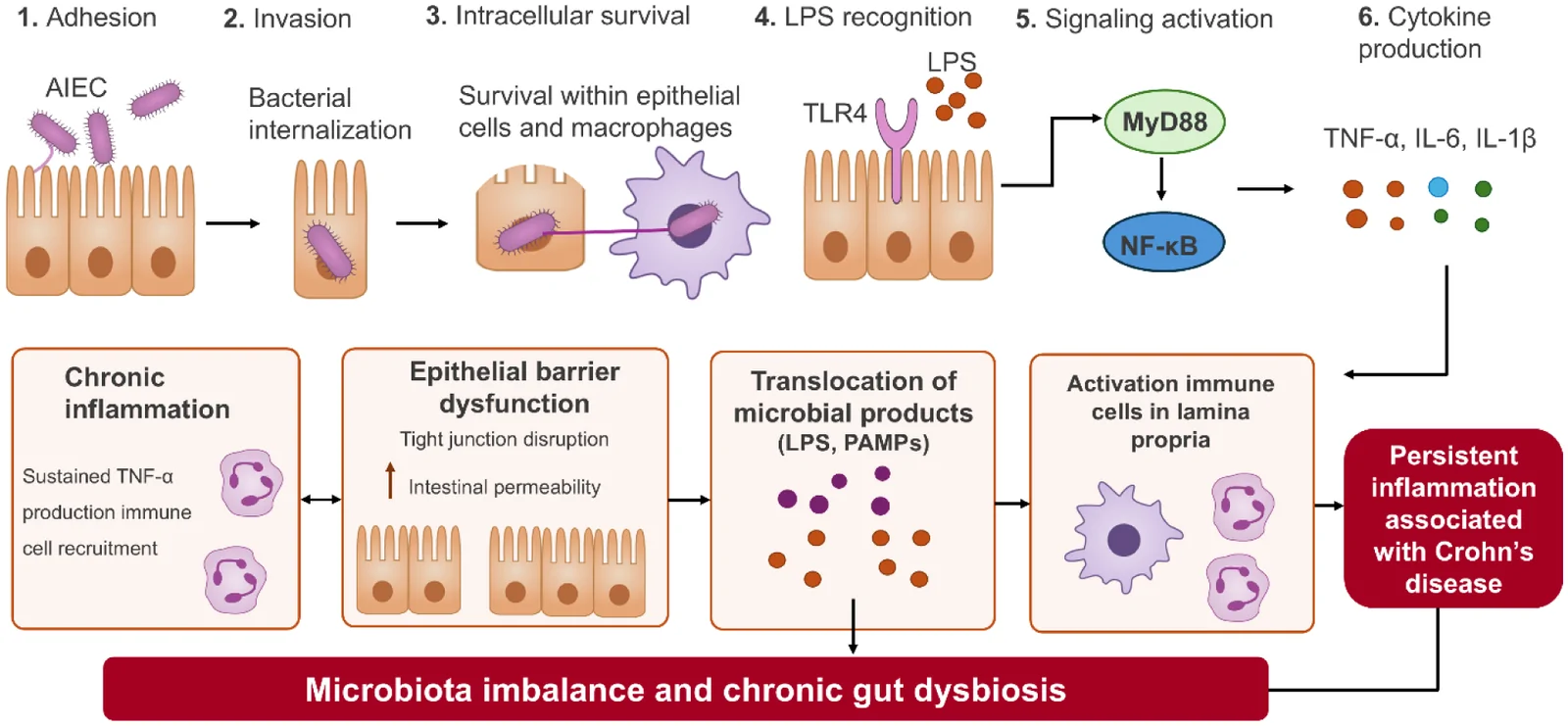
Mechanism of Adherent-invasive *Escherichia coli* (AIEC) in gut dysbiosis. AIEC contributes to gut dysbiosis through adhesion to and invasion of intestinal epithelial cells, followed by intracellular survival. Recognition of bacterial lipopolysaccharides (LPSs) activates the TLR4/NF-κB signaling pathway, inducing the production of pro-inflammatory cytokines, including TNF-α, IL-6, and IL-1β. Sustained inflammatory responses disrupt epithelial barrier integrity and increase intestinal permeability, facilitating the translocation of microbial products into the lamina propria and promoting immune-cell recruitment. Chronic inflammation and immune activation further weaken microbial homeostasis [55],[56],[58],[61],[62].

Prolonged inflammatory signaling disrupts epithelial barrier integrity through alterations in tight-junction proteins, resulting in increased intestinal permeability and translocation of microbial products into the lamina propria [58],[60],[62],[63]. These events further stimulate immune activation and establish a self-perpetuating inflammatory cycle. Chronic inflammation and barrier dysfunction are frequently accompanied by depletion of beneficial short-chain fatty acid (SCFA)-producing commensals and expansion of inflammation-adapted bacteria, thereby destabilizing microbial homeostasis and promoting persistent gut dysbiosis [55],[57],[58],[64].

#### 2.1.4. Fusobacterium nucleatum

*F. nucleatum* is an anaerobic Gram-negative bacterium increasingly recognized as a contributor to gut dysbiosis, chronic intestinal inflammation, and colorectal carcinogenesis [65]. As illustrated in Figure 4, one of its principal virulence mechanisms involves the adhesin FadA, which binds to E-cadherin on intestinal epithelial cells and activates the Wnt/β-catenin signaling pathway, thereby disrupting intestinal homeostasis and establishing a pro-inflammatory microenvironment [66],[67].

In addition to its direct effects on epithelial signaling, *F. nucleatum* exerts potent immunomodulatory activities. The bacterium stimulates antigen-presenting cells to produce IL-23, promoting Th17-cell differentiation and expansion and subsequent secretion of IL-17 [68]. *F. nucleatum* also promotes CRC cell proliferation and tumor development through activation of TLR4 signaling to NF-κB signaling and upregulation of microRNA-21 [69]. These immunomodulatory effects enhance chronic mucosal inflammation and support recruitment of inflammatory immune cells [70],[71]. Persistent activation of the IL-23/Th17 axis may contribute to tissue injury and sustain a pro-inflammatory environment that favors disease progression [70],[71].

Furthermore, *F. nucleatum* acts as a bridging organism within polymicrobial biofilms, facilitating microbial aggregation, mucosal colonization and potentially promoting interactions with the intestinal epithelium [72]. Biofilm formation facilitates persistent mucosal colonization and may contribute to epithelial barrier dysfunction and sustained inflammatory responses [72]. Concurrently, enrichment of *F. nucleatum* is frequently associated with depletion of beneficial short-chain fatty acid (SCFA)-producing bacteria, including *Faecalibacterium* and *Roseburia*, resulting in reduced butyrate production and impaired epithelial barrier function [73],[74]. The combined effects of epithelial signaling dysregulation, immune activation, biofilm formation, and loss of beneficial commensals establish a self-reinforcing cycle of chronic inflammation and gut dysbiosis that contributes to disease progression.

Beyond its role in epithelial signaling and biofilm-mediated colonization, *F. nucleatum* also contributes tumor-associated immune evasion and chemoresistance, two features increasingly recognized as important components of its involvement in colorectal carcinogenesis [75]. The fusobacterial autotransporter adhesin Fap2 binds the inhibitory checkpoint receptor TIGIT on natural killer cells (NK) and tumor-infiltrating T lymphocytes, inhibiting NK-cell cytotoxicity and T-cell activity and thereby shielding adherent bacteria and the adjacent tumor microenvironment from immune clearance [75]. The same adhesin mediates Fap2-dependent binding to Gal-GalNAc on colorectal adenocarcinoma cells, thereby establishing *F. nucleatum* within tumors and potentially reinforcing local immune escape [76],[77].

*F. nucleatum* enrichment has also been implicated in chemoresistance in CRC through several mechanisms, including activation of the Hippo signaling pathway with upregulation of BCL2, which suppress chemotherapy-induced pyroptosis; modulation of autophagy, and exosome-mediated transfer of resistance-conferring non-coding RNAs between tumor cells, diminishing the efficacy of agents such as 5-fluorouracil (5-FU) and oxaliplatin (OXA) , thereby contributing to post-chemotherapy disease recurrence [78],[79].

Tumoral *F. nucleatum* abundance has been associated with more advanced tumor stage, lymph node metastasis, and reduced overall survival, disease-free survival, and cancer-specific survival across cohort studies and meta-analyses, supporting its potential utility as a prognostic biomarker in addition to its proposed mechanistic role in disease progression and treatment resistance [72],[80],[81].

**Figure 4.**
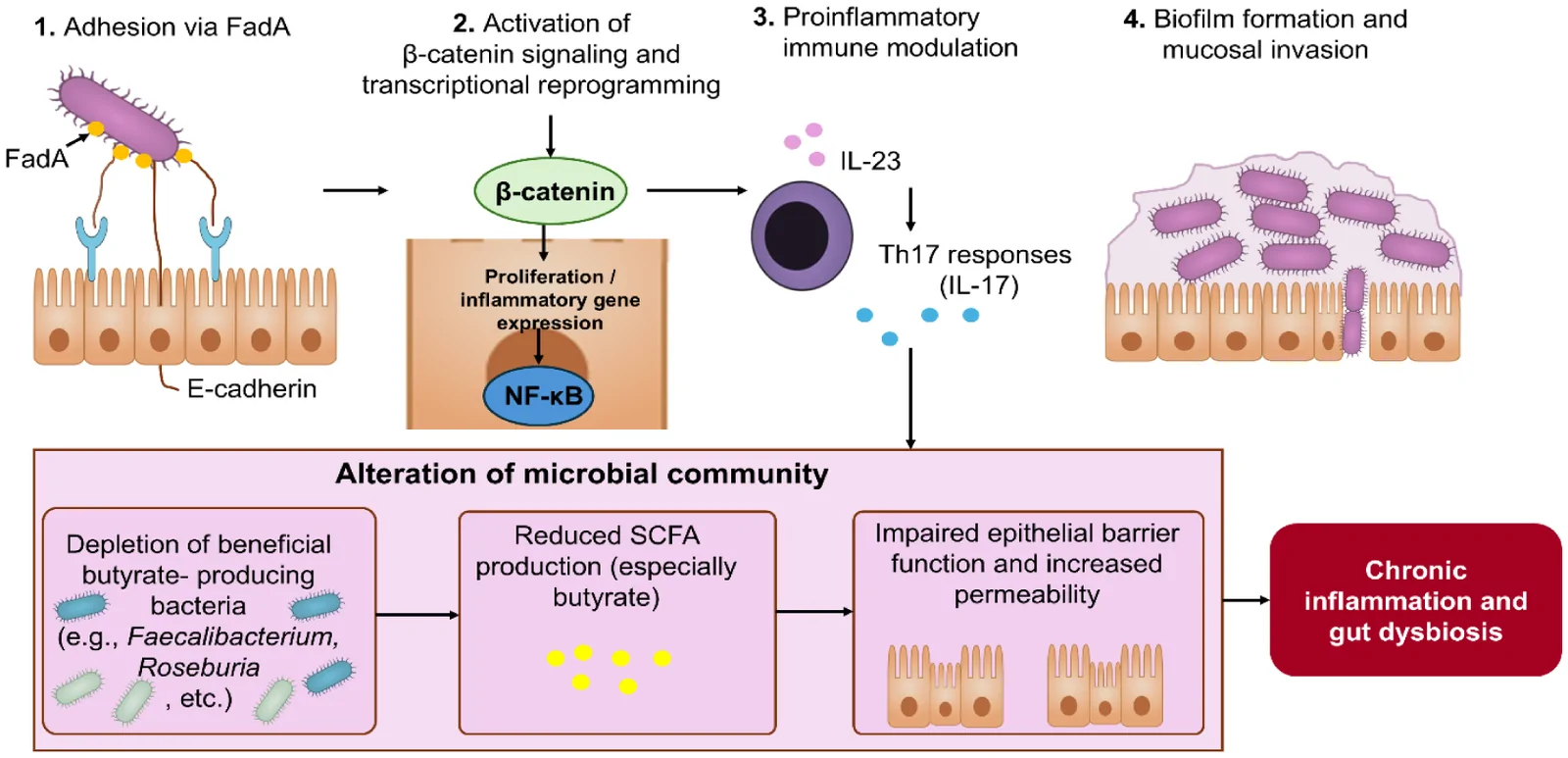
Mechanism of *Fusobacterium nucleatum* in gut dysbiosis and colorectal carcinogenesis. *F. nucleatum* promotes dysbiosis through multiple interconnected mechanisms. The adhesin FadA binds E-cadherin on intestinal epithelial cells, activating Wnt/β-catenin signaling and inducing the expression of genes involved in cellular proliferation, inflammation, and tumorigenesis [66],[67]. Furthermore, *F. nucleatum* promotes IL-23/Th17-mediated immune responses, leading to increased IL-17 production and establishment of a pro-inflammatory intestinal microenvironment [68],[70],[71]. In addition, the bacterium functions as a bridging organism within polymicrobial biofilms, enhancing microbial aggregation, mucosal colonization, and persistence within the intestinal niche [72]. Enrichment of *F. nucleatum* is associated with depletion of beneficial short-chain fatty acid (SCFA)-producing bacteria, including *Faecalbacterium* and *Roseburia*, resulting in reduced butyrate production, impaired epithelial barrier integrity, increased intestinal permeability, and sustained inflammation [73],[74]. Collectively, these processes contribute to chronic intestinal inflammation, microbial imbalance, and progression of colorectal disease [66]–[72],[74].

#### 2.1.5. Klebsiella pneumoniae

*K. pneumoniae* is an opportunistic pathobiont that can expand following disruption of colonization resistance and has been associated with IBD [82]–[84]. Intestinal colonization by *K. pneumoniae* promotes inflammatory responses through activation of innate immune pathways and epithelial dysfunction [82],[85]. Bacterial LPSs activates TLR4, leading to NF-κB signaling and induction of pro-inflammatory cytokines, including TNF-α, IL-6, and IL-1β [86],[87]. Sustained inflammation may impair epithelial barrier integrity and increase intestinal permeability, potentially favoring the expansion of facultative anaerobic Proteobacteria while contributing to the depletion of beneficial commensal microorganisms [82],[85]. In addition, *K. pneumoniae* serv as an important reservoir of antimicrobial resistance genes that can disseminate through horizontal gene transfer within the gut microbiota, facilitating the spread of multidrug resistance [82],[83],[85],[88]. Collectively, these mechanisms contribute to microbial imbalance, altered microbial metabolic activity, and persistent gut dysbiosis.

#### 2.1.6. Enterococcus faecalis

Although *E. faecalis* is a commensal member of the intestinal microbiota, it can become pathogenic under dysbiotic conditions and contribute to further microbial imbalance [89]. It contributes to dysbiosis through virulence factors that damage host tissues and promote chronic inflammation [89],[90]. *E. faecalis* can exploit disturbances in colonization resistance, particularly following antibiotic exposure, when protective commensals are depleted [89]. Under these conditions, *E. faecalis* can expand through biofilm formation, antimicrobial tolerance, and competitive interactions mediated by bacteriocins and other adaptive traits acquired through mobile genetic elements [89],[91],[92]. In parallel, virulence-associated factors such as GelE and SprE can compromise epithelial barrier integrity and promote inflammation, thereby creating ecological conditions that favor further enterococcal persistence and community imbalance [90],[93]. Adaptation to dysbiosis-associated changes in bile acid composition may further enhance its ability to persist in the disturbed gut [94].

#### 2.1.7. *Campylobacter spp*.

*Campylobacter* species, particularly *Campylobacter jejuni*, are important enteric pathogens that can contribute to gut dysbiosis through epithelial adhesion, invasion, and induction of mucosal inflammation [95]–[97]. Following ingestion, *Campylobacter* adheres to intestinal epithelial cells via adhesins such as CadF and FlpA, which facilitate colonization and mucosal invasion [95],[96]. *C. jejuni* subsequently produces cytolethal distending toxin (CDT) and induces host cells to produce pro-inflammatory cytokines and chemokines, including interleukin-8 (IL-8), thereby promoting the recruitment of neutrophils and other immune cells to the site of infection [95]–[98]. This inflammatory cascade contributes to epithelial injury, disruption of tight-junction integrity, and increased intestinal permeability [97]–[99]. In addition, *Campylobacter* infection is associated with alterations in microbial community structure, reduced abundance of beneficial commensal bacteria, and delayed microbiota recovery, which together may sustain post-infectious dysbiosis and impair restoration of intestinal homeostasis [20],[97],[100].

### 2.2. Antibiotic therapy and gut microbiota disruption

Antibiotics have revolutionized the treatment of bacterial infections and remain essential for managing many acute enteric diseases [101]. However, their broad-spectrum antimicrobial activity can profoundly disrupt the composition and function of the gut microbiota, frequently reducing microbial diversity, depleting beneficial commensal bacteria, and promoting the expansion of opportunistic pathogens, leading to gut dysbiosis [101]. The gut microbiota may partially recover after antibiotics are withdrawn, but repeated or prolonged exposure often results in persistent ecological disturbances, emphasizing the need for therapeutic strategies that preserve or restore microbial homeostasis during and after antimicrobial treatment [102].

Evidence from pediatric and adult populations highlights the long-term consequences of antibiotic-induced microbiota disruption [103]. In a longitudinal study involving 39 children, repeated antibiotic exposure during the first three years of life significantly reduced bacterial species and strain diversity, altered microbiota stability, and increased the abundance of antibiotic resistance genes [103]. Similarly, shotgun sequencing-based metagenomics analysis of healthy adults receiving a short course of broad-spectrum antibiotics demonstrated marked depletion of beneficial commensal bacteria together with transient expansion of opportunistic pathobionts and antibiotic-tolerant bacterial populations [104]. Although microbial diversity largely recovered within several weeks, several common bacterial taxa remained absent, indicating that even short-term antibiotic exposure can leave lasting ecological imprints on the gut microbiome [104]. These findings demonstrate that antibiotic treatment can induce both transient and persistent alterations in microbial community composition, increasing susceptibility to dysbiosis-associated diseases.

Recurrent *C. difficile* infection (rCDI) is a well-characterized consequence of antibiotic-induced gut dysbiosis. Repeated or broad-spectrum antibiotic exposure is strongly associated with reduced microbial diversity and increased risk of primary and recurrent CDI, while prior antibiotic exposure remains a major risk factor for recurrence and persistent dysbiosis [31],[105]–[108]. These effects arise because antibiotics disrupt colonization resistance and destabilize the resident microbial community, thereby increasing susceptibility to colonization by opportunistic and toxigenic pathogens [109].

Mechanistically, antibiotic-induced dysbiosis disrupts microbial bile acid metabolism by depleting commensal bile acid-transforming bacteria [33],[39]. This results in accumulation of primary and conjugated bile acids, such as taurocholate, that promote *C. difficile* spore germination, while reducing inhibitory secondary bile acids, including deoxycholate and lithocholate, that suppress spore germination and vegetative outgrowth, thereby facilitating *C. difficile* colonization and persistence [33],[39]. Alterations in bile acid composition have been strongly associated with rCDI, and restoration of secondary bile acid metabolism is considered an important mechanism underlying microbiome recovery after treatment [110]. Furthermore, repeated antibiotic exposure can exacerbate microbial depletion, delay microbiota recovery, and reinforce the dysbiotic environment that predisposes patients to recurrent infection [30],[38].

## 3. Microbiome-based therapeutic strategies for gut dysbiosis

The growing recognition of the gut microbiota as a key regulator of intestinal and systemic health has stimulated the development of microbiome-based therapeutic approaches aimed at restoring microbial balance. Among the most widely investigated strategies are fecal microbiota transplantation (FMT), live biotherapeutic products (LBPs), probiotics, and phage therapy, each of which employs distinct mechanisms to restore gut microbial balance and improve clinical outcomes.

### 3.1. Fecal Microbiota Transplantation

FMT involves the transfer of processed stool from a healthy donor into the gastrointestinal tract of a recipient with the aim of restoring microbial diversity and function [111]. FMT has emerged as one of the most effective microbiome-based therapies, particularly for rCDI [111],[112]. By introducing a complex and diverse microbial community, FMT can re-establish colonization resistance, restore microbiota-associated metabolic functions disrupted in conditions such as IBD and metabolic disorders, and suppress the expansion of opportunistic pathogens [32],[113]. Clinical studies have consistently demonstrated high efficacy, especially in rCDI, where FMT substantially outperforms conventional antibiotic therapy [111],[114].

Despite its remarkable efficacy, particularly in rCDI, FMT has several important limitations. Therapeutic outcomes may be influenced by donor-dependent variability, leading to inconsistent microbial engraftment and variable clinical responses among recipients. In addition, concerns remain regarding the potential transmission of infectious agents, antimicrobial resistance genes, and other undesirable microbial traits, despite increasingly stringent donor screening and pathogen-testing protocols [115],[116]. A notable example is the FDA safety alert issued while two patients died following FMT from a donor who was infected with Shiga toxin-producing *Escherichia coli* (STEC), a pathogenic group of *E. coli* that can cause severe bloody diarrhea [116].

Beyond rCDI, the beneficial effects of FMT in other conditions remain an active area of investigation including IBD, hepatic encephalopathy, and functional gastrointestinal disorders [112],[115],[117]. Although FMT is promising to be applied as a treatment strategy for several indications, further studies are required to establish its efficacy, safety, and long-term clinical benefits [112],[115],[117]. Consequently, substantial efforts are being directed toward the development of more defined and reproducible microbiome therapeutics that preserve the benefits of FMT while minimizing its limitations [23],[118]. These approaches include defined bacterial consortia and standardized LBPs, which may offer improved reproducibility and safety compared with unprocessed stool [23],[118].

### 3.2. Live biotherapeutic products

LBPs represent a next-generation of microbiome-based therapeutics composed of defined live microorganisms manufactured under controlled conditions and intended to confer a specific health benefit [118]. Unlike FMT, which transfers an entire donor-derived microbial community, LBPs consist of selected strains or microbial consortia designed to restore key ecological and metabolic functions of the gut microbiome [119]. This standardized composition can improve product consistency, safety, quality control, and regulatory oversight while reducing risks associated with donor-derived materials.

Advances in microbiome therapeutics have led to the regulatory approval of two new LBPs, RBX2660 (REBYOTA®) and SER-109 (VOWST®), against rCDI [120]. Although LBPs offer greater standardization and regulatory control than FMT, several limitations remain [121]. For example, achieving stable engraftment and long-term persistence in the highly competitive and individualized gut ecosystem is often difficult, and many administered strains do not establish durable colonization [121]. In addition, the optimal microbial composition, dosage form, formulation strategy, and manufacturing process remain incompletely defined, which may affect efficacy and reproducibility [122]–[124]. Safety considerations also remain important because the effects of administered microorganisms on the host depend on their specific biological characteristics and interactions within the complex gut ecosystem. The development of LBPs is further challenged by the complexity of microbial ecology and the limited understanding of how individual strains or consortia behave within the host. Therefore, a comprehensive understanding of host–microbe interactions and rigorous safety assessment throughout development and clinical treatment are essential [124]–[126]. These limitations underscore the need for improved formulation strategies, stronger quality-control standards, and long-term clinical studies to fully realize the therapeutic potential of LBPs.

### 3.3. Probiotics

Probiotics are live microorganisms that, when administered in adequate amounts, confer a health benefit to the host [127]. They have been investigated as a potential strategy for modulating gut dysbiosis by supporting microbial homeostasis [128]. Proposed mechanisms of action include competitive exclusion of pathogenic bacteria, production of antimicrobial compounds, enhancement of intestinal barrier integrity, modulation of host immune responses, and stimulation of beneficial microbial metabolites, including short-chain fatty acids (SCFAs) [128],[129]. A systematic review indicates that probiotic efficacy is highly strain- and disease-specific, and the selection of an appropriate probiotic therefore depends on the specific strain or strain combination and the target condition [130]. Evidence supports the use of specific probiotics for selected disorders, such as antibiotic-associated diarrhea (AAD) [130]. However, individual probiotic strains or combinations may provide benefits in particular preventive or therapeutic applications while showing limited or no efficacy in other acute or chronic conditions [130],[131].

A major limitation of probiotics is their inconsistent colonization and limited long-term persistence in the gastrointestinal tract. In healthy adults, probiotic supplementation does not always produce measurable or durable changes in microbiota composition, suggesting that ecological impact may be modest in some individuals [130],[132]–[134]. Moreover, host-specific microbiome features can influence probiotic engraftment and responsiveness, contributing to substantial inter-individual variability [132],[135]. While probiotics remain relevant tools for gut health research and selected clinical applications, their variable efficacy, limited persistence, and dependence on host-related factors highlight the need for more targeted and personalized microbiome-based interventions.

## 4. Bacteriophage (phage) therapy for gut dysbiosis

### 4.1. Phage therapy as a targeted intervention

Phage therapy represents a precision antimicrobial strategy that exploits the natural ability of phages to recognize, infect, and lyse susceptible bacterial hosts [136],[137]. Following adsorption to specific receptors on the bacterial surface, obligately lytic phages inject their genetic material, hijack the bacterial biosynthetic machinery to replicate, and ultimately lyse the host cell through the coordinated action of holins and endolysins [136],[137]. This highly selective life cycle distinguishes phages from broad-spectrum antibiotics, which can substantially disrupt both pathogenic and beneficial bacterial populations and thereby exacerbate gut dysbiosis [26]–[28].

The remarkable host specificity of bacteriophages enables the selective reduction of disease-associated bacterial populations [26]–[28]. Consequently, phage therapy is particularly attractive for treating dysbiosis driven by the overgrowth of specific pathogens [26]–[28]. Its high host specificity enables targeted pathogen reduction with minimal collateral effects on beneficial members of the gut microbiota, thereby preserving microbial diversity and ecological stability compared with conventional broad-spectrum antibiotic therapy [26]–[28]. By reducing pathogen burden, phages can indirectly restore colonization resistance, decrease intestinal inflammation, preserve epithelial barrier integrity, and promote the re-establishment of beneficial microbial communities [27],[28]. Phage-mediated removal of selected bacterial populations can induce predictable changes in microbial community composition and metabolic activity, thereby reshaping ecological interactions within the gut microbiome [12].

Phage therapy therefore represents a complementary microbiome-based therapeutic strategy alongside FMT, LBPs, and probiotics, although these approaches differ substantially in their mechanisms, advantages, and limitations. Table 1 summarizes the major differences among FMT, LBPs, probiotics, and phage therapy as therapeutic strategies for gut dysbiosis.

**Table 1.** Comparison of microbiome-based therapeutic strategies for the management of gut dysbiosis.

Therapeutic approach	Mechanism	Advantages	Key limitations	Clinical evidence	Reference
Fecal Microbiota Transplantation (FMT)	Restores microbial diversity and function through transfer of a donor-derived microbial community	Highly effective for recurrent *Clostridioides difficile* infection (rCDI); can restore microbial diversity, colonization resistance, and metabolic functions.	Donor variability, risk of pathogen transmission risk, limited standardization, variable engraftment, and uncertain long-term safety.	Strong evidence for rCDI; growing evidence for IBD and other microbiome-associated disorders, although efficacy remains variable across indications.	[21],[111],[112],[114],[115]
Live Biotherapeutic Products (LBPs)	Introduce defined live microbial strains or consortia to restore key ecological and metabolic functions of the gut microbiome	Defined composition, improved manufacturing consistency, enhanced quality control, and greater regulatory oversight than FMT	Limited engraftment and long-term persistence, strain-specific efficacy, manufacturing complexity, and incomplete understanding of optimal microbial composition	Moderate to strong evidence for rCDI; FDA-approved products such as Rebyota® and Vowst® reduce the risk of CDI recurrence; however, evidence for other gastrointestinal and metabolic disorders remains limited	[23],[118]–[120],[125]
Probiotics	Administration of specific live microorganisms to improve microbial composition, enhance epithelial barrier function, and modulate host immune responses	Generally accessible, relatively, inexpensive, easy to administer, and beneficial for selected conditions, particularly antibiotic-associated diarrhea and some gastrointestinal disorders	Strain-specific efficacy, transient colonization, inconsistent clinical outcomes, and limited long-term persistence	Moderate and strain-specific evidence; strongest support for antibiotic-associated diarrhea and selected gastrointestinal disorders, whereas efficacy remains inconsistent across many dysbiosis-associated conditions	[130],[132]–[134],[138]
Phage Therapy	Selectively targets and lyses disease-associated bacterial pathogens using lytic bacteriophages, potentially minimizing disruption of commensal microbiota	Precision targeting, potential activity against multidrug-resistant pathogens, reduced collateral microbiome disruption, and compatibility with personalized therapy	Narrow host range, emergence of phage resistance, host immune clearance, delivery challenges, manufacturing and regulatory barriers, and limited clinical evidence	Limited clinical evidence;most studies remain *in vitro*, *ex vivo*, or preclinical animal-model stage. Human evidence consists mainly of compassionate-use cases, case reports, and early-phase clinical trials, with relatively few randomized controlled trials	[26]–[28],[136],[139]

### 4.2. Phage therapy for restoring gut dysbiosis

Phage therapy offers a highly determined approach for modulating microbial communities. Unlike broad-spectrum antibiotics, which often indiscriminately eliminate pathogenic and beneficial microorganisms, phages can selectively reduce disease-associated bacterial populations while preserving the overall structure and function of the commensal microbiota [26]–[28]. Phage therapy focuses on the selective removal of disease-associated bacterial populations that drive dysbiosis. It can act synergistically with microbiota restoration approaches by reducing pathogen burden, alleviating inflammation, and creating ecological space for beneficial microorganisms to re-establish colonization and function within the gut ecosystem [10],[12],[26]–[28]. Phage therapy has emerged as a promising precision-based strategy for selectively targeting bacterial pathogens associated with gut dysbiosis while minimizing disruption of beneficial commensal microbiota. Several studies have demonstrated the isolation, characterization, and preclinical effect of phages against key dysbiosis-associated pathogens, which are summarized in Table 2.

**Table 2.** Bacteriophage applications targeting gut dysbiosis-associated pathogens.

Year	Pathogen/target	Phage(s)	Model	Experimental model	Stage	Main Finding	Key Limitations	Reference
2005	*Clostridioides difficile*	φC2, φC5, φC6, φC8	*In vitro*	Isolation and characterization of temperate *C. difficile* phages	Phage characterization	Identified and characterized temperate bacteriophages infecting *C. difficile*	Temperate phages; no therapeutic efficacy evaluation	[140]
2006	*Clostridioides difficile*	ΦCD119	*In vitro*	Genomic and molecular characterization of temperate bacteriophage ΦCD119	Phage characterization	Characterized the genome, lysogeny-associated genes, and biological properties of ΦCD119, providing foundational knowledge for *C. difficile* phage research	Descriptive study; no therapeutic efficacy or treatment model	[141]
2007	*Clostridioides difficile*	Eight temperate bacteriophages isolated from *C. difficile* strains	*In vitro*	Morphological and genetic characterization	Phage characterization	Demonstrated substantial morphological and genetic diversity among temperate *C. difficile* phages	Temperate phages; no therapeutic efficacy evaluation	[142]
2007	*Clostridioides difficile*	φC2	*In vitro*	Complete genome sequencing and comparative genomic analysis	Phage characterization	Characterized the complete genome of temperate phage φC2 and demonstrated genetic relatedness to φCD119 and CD630 prophages, improving understanding of *C. difficile* phage biology	Temperate phage; descriptive genomic study with no therapeutic efficacy evaluation	[143]
2017	AIEC (*E. coli* LF82)	Three-phage cocktail	*In vivo/Ex vivo*	CEABAC10 mouse model and Crohn's disease ileal biopsy homogenates	Preclinical efficacy	Significantly reduced intestinal AIEC colonization and alleviated colitis symptoms.	Preclinical study; host-range limitations and potential phage resistance require further evaluation	[144]
2019	*Fusobacterium nucleatum*	FNU1	*In vitro*	Genomic characterization and biofilm-disruption assays using laboratory-grown *F. nucleatum* biofilms	Preclinical proof-of-concept	Significantly disrupted established *F. nucleatum* biofilms, demonstrating potential for targeted control of *F. nucleatum*–associated diseases	Limited to laboratory biofilm models; no animal studies, polymicrobial biofilm validation, or clinical evaluation	[145]
2022	AIEC (*E. coli* LF82 and clinical AIEC strains)	EcoActive™ (7-phage cocktail)	*In vitro/In vivo*	Host-range testing against 210 AIEC strains and DSS-induced colitis mouse model colonized with LF82SK	Preclinical efficacy	Spared representative commensal bacteria, did not induce dysbiosis in healthy mice, and repeated administration significantly reduced colitis severity in AIEC-colonized mice	Preclinical study; efficacy required repeated dosing and remains unproven in humans	[146]
2022	IBD-associated *Klebsiella pneumoniae* (Kp2 clade, ST323)	5-phage cocktail	*In vitro/In vivo/Ex vivo/* Phase I clinical	Phage selection and optimization, multiple mouse colitis models, SHIME human gut simulator, and healthy volunteer study	Early clinical translation	Suppressed IBD-associated *K. pneumoniae*, reduced intestinal inflammation and disease severity in mouse models, and demonstrated gastrointestinal survival, safety, and microbiota preservation in healthy volunteers	Clinical efficacy in in patients with IBD has not been established; Phase I primarily assessed safety and phage viability rather than therapeutic efficacy	[147]
2022	*Salmonella enterica* (multiple serovars including *S. Enteritidis and S. Typhimurium)*	3-phage cocktail	*In vitro/In vivo*	*In vitro* lysis assays and oral administration of a phage cocktail to broilers under commercial farm conditions	Preclinical efficacy (field pilot study)	*Salmonella* populations reduced by ~4 log units *in vitro*, no detectable cytotoxicity.	Pilot study with limited sample size and single-farm evaluation; large-scale commercial efficacy and long-term outcomes require further investigation	[148]
2023	F18 enterotoxigenic *Escherichia coli* (ETEC)	vB_EcoS_ULIM2, vB_EcoM_ULIM3, vB_EcoM_ULIM8, vB_EcoM_ULIM9	*In vitro/In vivo*	Phage characterization, *Galleria mellonella* infection model, and static piglet gut microbiota (SHIME-derived) system	Preclinical proof-of-concept	Significantly improved survival of infected *G. mellonella* larvae and showed minimal impact on piglet gut microbiota composition and SCFA production	No pig infection model or clinical validation; efficacy demonstrated only in an insect model and static microbiota system	[149]
2025	*Salmonella enterica serovar Typhimurium* (STm)	Three *Salmonella* Phages (LPST83, LPST94, and LPST153)	*In vitro/ In vivo*	Phage cocktail screening, hydrogel microsphere delivery system, and STm-induced murine colitis model	Preclinical efficacy	Reduction of intestinal STm burden by ~2000-fold, decreased pro-inflammatory cytokine production, alleviated colitis symptoms, and improved gut microbiota diversity while preserving beneficial bacterial taxa	Mouse study only; no human clinical validation; long-term efficacy, resistance dynamics, and scalability of the delivery platform require further investigation	[150]

### 4.3. Limitations of phage therapy

#### 4.3.1. Development of phage resistance

Similar to antibiotics, bacteriophages exert selective pressure that can promote bacterial evolution and the emergence of resistance mechanisms [151]. Bacteria can evade phage infection through modification or loss of surface receptors required for phage adsorption, thereby preventing phage attachment to the bacterial cell [151]. Additional defense mechanisms include restriction-modification systems that degrade invading phage DNA and CRISPR-Cas adaptive immune systems that recognize and cleave previously encountered phage genomes [151]. Some bacteria also employ abortive infection systems, in which infected cells undergo programmed cell death to prevent phage replication and limit phage dissemination within the bacterial population [151]. These defense mechanisms can substantially reduce the efficacy of single-phage therapy and contribute to treatment failure if not adequately addressed [136],[151].

Several strategies have been proposed to overcome phage resistance. Phage cocktails containing multiple lytic phages that target distinct bacterial receptors can reduce the likelihood of resistance emerging simultaneously against all phages [26],[151],[152]. Likewise, combining phages with conventional antibiotics has demonstrated synergistic antibacterial activity and, in some settings, suppression of resistant subpopulations; however, choosing the phage type, antibiotics, concentrations, and dose must be carefully optimized [153],[154]. Engineering phages to broaden their host range may improve bacterial coverage and reduce the likelihood of escape by resistant variants [152],[155].

#### 4.3.2. Host immune responses

The host immune system represents another important factor influencing the success of phage therapy. Following administration, innate, and adaptive immune mechanisms may recognize phages as foreign biological particles [27],[156]. This immune recognition may result in the production of neutralizing antibodies that reduce phage activity during repeated dosing [157]. In addition, complement activation, phagocytosis by macrophages, and uptake by the mononuclear phagocyte system may accelerate phage clearance, thereby shortening their persistence within the gastrointestinal tract [27],[157].

Within the intestinal environment, mucus layers may physically retain bacteriophages, while innate immune responses and inflammatory conditions can alter phage distribution and accessibility to bacterial targets [156],[158]. Although these interactions may occasionally enhance mucosal protection through the natural bacteriophage adherence to mucus (BAM) model, excessive immune clearance could reduce therapeutic effectiveness, particularly in chronic diseases requiring repeated administration [156],[158]. Consequently, optimization of dosing frequency, delivery vehicles, and encapsulation technologies will be important for maximizing phage persistence and therapeutic efficacy.

#### 4.3.3. Horizontal gene transfer and lysogenic conversion

Safety concerns surrounding phage therapy extend beyond antibacterial efficacy. Temperate bacteriophages can integrate into bacterial chromosomes as prophages and promote horizontal gene transfer through lysogenic conversion and specialized transduction. As a result, antimicrobial resistance genes, toxin genes, and other virulence determinants may be disseminated among bacterial populations, potentially enhancing pathogen fitness and disease severity [159]–[161]. Therefore, therapeutic development has increasingly prioritized lytic bacteriophages that lack lysogeny-associated functions. Whole-genome sequencing of candidate phages is now considered essential to exclude integrases, repressors, virulence genes, antimicrobial resistance determinants, and other undesirable genetic elements before clinical use. Such genomic screening is an important component of contemporary phage development and quality-assessment pipelines [162]–[166].

#### 4.3.4. Effects on non-target microbial populations

Although phages are substantially more specific than broad-spectrum antibiotics, they may exert indirect effects on non-target microbial populations. Moreover, eliminating dominant bacterial taxa can alter nutrient availability, metabolite production, oxygen gradients, and inflammatory signaling, thereby generating secondary ecological shifts within the gut microbiome [10],[12],[27],[28]. In addition, phage cocktails with broader host ranges may occasionally affect closely related commensal organisms with similar surface receptors [163],[166]. Therapeutic phage administration may also transiently reshape the intestinal virome and influence gene transfer dynamics within resident bacterial communities [10],[12],[15],[27],[28]. Accordingly, longitudinal metagenomic and metavirome analyses should accompany future clinical studies to assess ecological consequences beyond pathogen reduction and to ensure preservation of overall microbiome stability [10]–[12],[15].

#### 4.3.5. Manufacturing, scalability, and quality control

Therapeutic bacteriophage products should be manufactured under GMP/cGMP conditions to ensure consistent quality, safety, and efficacy [167],[168]. GMP requires comprehensive documentation, qualified personnel, validated processes, and robust quality management systems throughout the product lifecycle [167],[168]. Large-scale production of clinical-grade phages remains challenging because phages must be propagated in bacterial hosts and then extensively purified to remove endotoxins, host-cell debris, nucleic acids, and other contaminants while preserving infectivity [162],[164],[168],[169]. Achieving batch-to-batch reproducibility, standardized potency, and long-term stability remains difficult, particularly for personalized phage cocktails that require isolate-specific selection, susceptibility testing, and individualized manufacturing [162],[166],[170]. Therefore, validated quality-control assays and oral delivery strategies that protect phages from gastric acidity and digestive enzymes remain major priorities for clinical translation [169],[171],[172].

#### 4.3.6. Cost-effective considerations

The economic feasibility of phage therapy remains an important challenge for widespread implementation. Personalized therapeutic approaches that require patient-specific phage isolation, genomic characterization, susceptibility testing, and customized manufacturing are likely to increase treatment costs [162],[164],[166]. In addition, the establishment of phage banks, GMP-compliant production facilities, comprehensive quality-control programs, and regulatory compliance may further increase development and production expenses, potentially limiting large-scale implementation [164],[165],[169]–[171],[173]. Formal health-economic analyses and cost-effectiveness studies are therefore needed to identify clinical scenarios in which phage therapy may provide economic advantages over existing antimicrobial or microbiome-based interventions [170],[173],[174]. Increased governmental funding, stronger academic-industry partnerships, and international collaborative research programs may help reduce development costs and accelerate clinical translation of phage therapeutics [170],[173],[174].

#### 4.3.7. Regulatory approval and clinical implementation

Regulatory approval and widespread clinical implementation remain significant challenges, although regulatory agencies have established guidance documents, scientific workshops, clinical trial pathways, and expanded-access programs to support phage therapy development. However, a globally harmonized framework remains lacking, particularly for personalized and adaptive phage products that do not fit conventional pharmaceutical regulations [165],[168],[170],[174]–[176]. Regulatory challenges include phage characterization, manufacturing consistency, quality control, and the approval of patient-specific or adaptive phage preparations [165],[168],[170],[174]–[176]. To facilitate clinical translation, regulatory reforms have been proposed to support more flexible approval pathways, adaptive clinical trial designs, and standardized requirements for phage production and evaluation [165],[174],[175],[177]. International harmonization of regulatory standards would further accelerate the development and accessibility of safe and effective phage-based therapeutics [165],[174],[175],[177].

#### 4.3.8. Critical evaluation of evidence quality and translational readiness

Evidence supports bacteriophage therapy as a promising precision approach for managing gut dysbiosis, but most studies remain preclinical. Existing data from *in vitro*, *ex vivo*, and animal models show that phages can selectively target dysbiosis-associated pathogens. Some studies also demonstrate reduced inflammation, biofilm disruption, improved microbiota composition, and restoration of microbial homeostasis.

However, the overall evidence base remains limited because many studies focus on phage discovery, host-range testing, genomic characterization, or early proof-of-concept experiments rather than rigorous therapeutic validation. Key limitations include simplified models, small animal studies, narrow phage host range, need for phage cocktails, incomplete dosing data, limited pharmacokinetic knowledge, and insufficient assessment of long-term microbiome and immune effects.

Translational readiness varies by pathogen. Phage approaches targeting AIEC, *E. faecalis*, *Salmonella* spp., and IBD-associated *K. pneumoniae* have advanced into more sophisticated models, including murine disease systems, humanized microbiota models, *ex vivo* human-derived systems, and early clinical safety evaluation. In contrast, phage studies targeting *Campylobacter* spp., *C. difficile*, and *F. nucleatum* have generally remained at thecharacterization, cocktail development, or early preclinical proof-of-concept models.

Overall, phage therapy has progressed from basic phage discovery toward targeted microbiome intervention, but successful clinical translation will require standardized manufacturing, validated quality-control assays, rational cocktail design, longitudinal microbiome and metavirome monitoring, and well-designed randomized clinical trials.

## 5. Future perspectives

Conventional microbiome-based interventions, including FMT, LBPs, and probiotics, have demonstrated therapeutic potential for restoring microbial balance; however, their efficacy remains constrained by donor variability, limited engraftment, and considerable inter-individual variation in treatment response [21],[23],[118]. These limitations have stimulated growing interest in precision microbiome therapies capable of selectively targeting disease-associated microorganisms while preserving beneficial commensal populations. Phage therapy is increasingly being investigated as a precision approach toward precision microbiome medicine, and several emerging technologies may further accelerate its clinical translation.

### 5.1. Artificial intelligence-guided phage–host prediction and rational cocktail design

Advances in artificial intelligence (AI), machine learning, and bioinformatic modeling have enabled the development of computational approaches for predicting phage–host interactions using genomic sequences, receptor-binding protein characteristics, and bacterial susceptibility data [178]–[180]. These approaches may substantially accelerate phage discovery and selection compared with conventional culture-based host-range screening methods, thereby facilitating the development of more efficient therapeutic phage preparations [163],[166],[181]. Nevertheless, the performance of current phage-host prediction tools remain strongly dependent on the availability of genomic reference databases and experimentally validated host–phage interaction datasets [179],[180]. Consequently, predictive accuracy and validation are generally higher for well-characterized bacterial taxa, whereas coverage remains uneven across many clinically relevant microorganisms in addition to host–phage datasets and pathogen-specific validation studies will be required before AI-guided phage selection can be broadly applied to dysbiosis-associated pathogens [179],[180].

Beyond host-range prediction, AI-driven integration of metagenomic, metatranscriptomic, metabolomic, and clinical datasets may facilitate identification of disease-associated microbial signatures and improve our understanding of host–microbiome interactions [182],[183]. These approaches have demonstrated potential for disease classification, biomarker discovery, risk stratification, and prediction of treatment responses, supporting the broader development of precision microbiome medicine [182],[183]. As microbiome datasets continue to expand, integration of AI-assisted microbiome analytics with advances in phage-host prediction may provide additional opportunities to support future microbiome-targeted therapeutic strategies.

### 5.2. CRISPR-armed and genetically engineered phages

One promising approach involves CRISPR-armed phages that deliver programmable CRISPR systems into target bacteria, enabling highly selective elimination of specific bacterial pathogens. Experimental study has demonstrated successful *in vitro and in vivo* targeting of *C. difficile* using bacteriophage-delivered CRISPR-Cas3, resulting in enhanced bacterial killing, reduced pathogen burden, and decreased disease severity [184]. These findings highlight the potential of engineered CRISPR-phage therapeutics as precision antimicrobials for targeted bacterial control while potentially minimizing disruption of beneficial members of the gut microbiota.

Additionally, genetic engineering techniques can be used to modify phage receptor-binding proteins, thereby expanding host range or redirecting phage specificity toward clinically relevant bacterial strains [152],[178],[185]. Furthermore, genome editing enables the removal of lysogeny-associated genes, virulence factors, antimicrobial resistance determinants, and other undesirable genetic elements, thereby improving the safety profile of candidate therapeutic phages [159],[161],[186]. These technologies may be particularly valuable for pathogens such as *C. difficile* and *F. nucleatum*, where many characterized phages are temperate rather than strictly lytic [142],[145]. In the future, engineered phages may also serve as targeted delivery vehicles for therapeutic payloads, anti-biofilm agents, or precision gene-editing systems within the gastrointestinal tract.

### 5.3. Microbiome digital twins and personalized phage therapy

Genome-scale metabolic modeling and microbiome “digital twin” platforms are increasingly being used to simulate microbial ecosystem dynamics and predict patient-specific responses to therapeutic interventions [187]–[190]. By integrating microbiome composition, microbial metabolism, host characteristics, and clinical data, these computational frameworks may enable prediction of how individual phages or phage cocktails will interact with a patient's resident microbiota [187],[189],[191]. Such models could potentially support optimization of phage selection, dosing strategies, treatment duration, and combination therapies involving probiotics, LBPs, or FMT.

Developments in explainable AI may further enhance these approaches by providing mechanistic insight into phage-mediated microbiome modulation rather than relying solely on empirical predictions [183],[192]. Ultimately, integration of AI-guided phage selection, synthetic biology, microbiome modeling, and personalized medicine approaches may facilitate the transition of phage therapy from experimental proof-of-concept studies toward clinically applicable precision microbiome interventions.

### 5.4. Integration with microbiome-restoration strategies

Phage therapy is increasingly viewed as a complementary approach that can be integrated with microbiome-restoration strategies, such as probiotics and other microbiome-based interventions [25],[193]. By selectively reducing pathogen burden, bacteriophages may create ecological niches that facilitate recolonization by beneficial microorganisms and support restoration of microbial homeostasis.

Studies have demonstrated the potential benefits of combination therapies. For example, phage cocktails administered together with microcin-producing probiotic *Escherichia coli* strains significantly reduced intestinal colonization by multidrug-resistant *E. coli* ST131 [194]. Similarly, combined phage–probiotic therapy improved intestinal barrier function, modulated gut microbial composition, and reduced multidrug-resistant *Salmonella* colonization in experimental animal models [195]. These findings suggest that phages and beneficial microorganisms may act synergistically by simultaneously suppressing pathogens and promoting recovery of beneficial microbiota.

### 5.5. Translational and regulatory roadmap

Although bacteriophage therapy has demonstrated considerable preclinical potential for the management of gut dysbiosis, successful clinical translation will require the resolution of several scientific, manufacturing, and regulatory challenges [164],[172]. Key priorities include the establishment of standardized phage production processes, validated quality-control and potency assays, optimized delivery systems for gastrointestinal administration, comprehensive safety assessments, and adequately powered randomized clinical trials to demonstrate clinical efficacy and reproducibility [164],[172].

Regulatory frameworks must also address issues unique to phage therapy, including phage characterization, genomic stability, batch-to-batch consistency, adaptive cocktail reformulation, and long-term safety monitoring. Particular attention should be given to potential host immune responses, phage resistance development, and the risk of unintended horizontal gene transfer. Although regulatory agencies have begun developing guidance for phage-based medicinal products, important challenges remain in adapting conventional pharmaceutical regulations to personalized and rapidly evolving phage preparations [165],[168],[170],[174],[175],[177],[193].

Continued advances in phage engineering, genome characterization, host-range prediction, and manufacturing technologies are expected to improve the safety, efficacy, and scalability of phage-based therapeutics [174]. In parallel, longitudinal microbiome and virome studies will be essential for understanding the long-term ecological consequences of phage-mediated bacterial depletion and for ensuring durable restoration of microbial homeostasis [10],[15],[193].

Overall, emerging developments in AI, synthetic biology, systems biology, and microbiome science are creating new opportunities for precision phage therapy. As these technologies continue to mature, bacteriophage-based therapeutics may become an important component of personalized microbiome-targeted strategies for the prevention and management of gut dysbiosis and its associated diseases [180],[182],[183].

## 6. Conclusion

Gut dysbiosis is increasingly recognized as a central contributor to a wide range of gastrointestinal, metabolic, inflammatory, and systemic diseases. Pathogenic and opportunistic bacteria, including *C. difficile*, adherent-invasive *Escherichia coli* (AIEC), *Salmonella Typhimurium*, *F. nucleatum*, *K. pneumoniae*, *E. faecalis*, and *Campylobacter* spp., can disrupt microbial homeostasis through diverse yet interconnected mechanisms, including loss of colonization resistance, epithelial barrier dysfunction, chronic immune activation, metabolic alterations, and depletion of beneficial commensal microorganisms.

To control these disturbances, several microbiome-based therapeutic strategies have been developed. FMT, LBPs, and probiotics primarily aim to restore microbial balance through the introduction of beneficial microorganisms or microbial communities. In contrast, bacteriophage therapy offers a fundamentally different precision-based approach by selectively targeting disease-associated bacterial populations while with the potential to preserve a greater proportion of commensal microbiota than broad-spectrum antibiotics. This high degree of specificity represents a major theoretical advantage over conventional broad-spectrum antimicrobial interventions.

The evidence reviewed in this article demonstrates that bacteriophages can selectively target a broad range of dysbiosis-associated pathogens and, in many experimental models, reduce inflammation, suppress biofilm formation, promote recovery of community structure, and improve intestinal homeostasis. However, most available evidence remains derived from *in vitro*, *ex vivo*, and animal studies, and relatively few phage-based interventions have progressed to rigorous human clinical evaluation. Important challenges remain, including the emergence of phage resistance, host immune clearance, limitations associated with narrow host range, the safety implications of temperate phages, manufacturing standardization, delivery optimization, and evolving regulatory requirements.

Despite these challenges, continued advances in phage engineering, genomic screening, artificial intelligence-guided phage selection, systems-level microbiome modeling, and regulatory science are expanding the potential of bacteriophage-based therapeutics. Future progress will depend on well-designed clinical trials, standardized manufacturing frameworks, and comprehensive evaluation of long-term ecological effects on the gut microbiome and virome.

Overall, bacteriophage therapy represents a promising addition to the growing portfolio of microbiome-directed interventions. Although significant scientific, clinical, and regulatory hurdles remain, its precision-targeting capability positions phage therapy as a potentially valuable component of future personalized strategies for restoring gut microbial homeostasis and managing dysbiosis-associated disease.

## Use of AI tools declaration

The authors declare they have not used Artificial Intelligence (AI) tools in the creation of this article.
